# Explanation and Correction

**Published:** 1899-09

**Authors:** 


### EXPLANATION AND CORRECTION.

In the August number of this journal appeared an editorial in which criticism was made as to the disposition of proceedings. In this criticism was included the oral section of the National Medical Association. The secretary of this organization, Dr. Eugene S. Talbot, writes that, "According to the law of the Association, all papers read before the section are property of the Association, and must be turned over to the Publication Committee as soon as they are received by the secretary of the section. . . . I have never . . . in any way given any journal the preference in obtaining abstracts of papers."

We are glad of the opportunity of placing the oral section in a correct position. The idea has been entertained that there has been a preference. The main contention in the editorial in question was, that all associations, whether local, State, or national, should publish their own proceedings. This it seems the oral section of the National Medical Association does, and it should receive due credit therefore.

